# An Attempt of a New Strategy in PRV Prevention: Co-Injection with Inactivated *Enterococcus faecium* and Inactivated Pseudorabies Virus Intravenously

**DOI:** 10.3390/v15081755

**Published:** 2023-08-17

**Authors:** Yuan Cui, Libo Huang, Jinlian Li, Gang Wang, Youfei Shi

**Affiliations:** 1 College of Animal Science and Veterinary Medicine, Shandong Agricultural University, Tai’an 271018, China; cuiyuan1995123@163.com (Y.C.); huanglibo123@126.com (L.H.); 2 College of Biology and Brewing Engineering, Taishan University, Tai’an 271021, China; lijinlian1979@163.com

**Keywords:** pseudorabies virus, *Enterococcus faecium*, immunization, intravenous

## Abstract

Pseudorabies virus (PRV) is one of the causative agents of common infectious diseases in swine herds. *Enterococcus faecium* is a probiotic belonging to the group of lactic acid bacteria and has excellent immunomodulatory effects. Vaccine immunization is an important approach to prevent animal diseases in the modern farming industry, and good immunization outcomes can substantially reduce the damage caused by pathogens to animals, improve the quality of animals’ lives, and reduce economic losses. In the present study, we showed that inactivated *E. faecium* and inactivated PRV when co-injected intravenously significantly reduced the mortality of mice after inoculation with PRV. The inactivated *E. faecium* + inactivated PRV intravenous injection group induced more production of Th cells and Tc cells. Additionally, the inactivated *E. faecium* + inactivated PRV intravenous injection group showed higher concentrations of cytokines (IFN-γ and IL-10) and induced higher antibody production. Thus, the co-injection of inactivated *E. faecium* and inactivated PRV could remarkably prevent and control the lethality of PRV infection in mice, which is a critical finding for vaccination and clinical development.

## 1. Introduction

Pseudorabies virus (PRV), the causative agent of pseudorabies, is a swine herpesvirus that belongs to the genus *Varicellovirus* in the subfamily *Alphaherpesvirinae* of the family *Herpesviridae* [1]. PRV is lethal to many domestic and wild animals, for example, cattle, sheep, and dogs, while pigs are the natural host of this virus. PRV was first reported in swine herds of China in 1956, and since 2012, several PRV variants have been detected, which has led to massive economic losses of swine farms in China. A serious concern is that PRV can be transmitted from pigs to humans, with neurological dysfunction as a typical symptom, and the disease can eventually cause vegetation or death in humans [2,3,4,5].

PRV is globally distributed and poses a high risk to livestock and human health; furthermore, vaccination is the most effective and economical approach to prevent infectious diseases [6,7,8]. However, because of immune evasion by the virus and the immunologically silent nature of its latency, the development of effective and safe PRV vaccines remains challenging. 

Vaccine adjuvants are substances that can modulate antibody and cell-mediated adaptive immune responses, reduce the antigen dose used, and reduce the number of immunizations based on the highest immunity achieved for animals [9,10]. Probiotics play a key role in shaping the maturation and activity of the immune system in animals. Several field studies have shown the positive effects of probiotics on animal growth performance and their immune system [11,12]. Probiotics mainly activate the innate immune response. This feature of probiotics is the key for the subsequent stimulation of an adaptive immune response [13]. Probiotics are used as vaccine adjuvants to further enhance the immunogenicity and effectiveness of live vaccines [14]. As a protective antigen delivery carrier, probiotics have the advantages of high safety, and oral or nasal mucosal administration can induce mucosal immunity, humoral immunity, and cellular immunity [15]. Following the administration through the oral route, the probiotics enter the gut and induce a systemic immune response by causing the release of cytokines via mucosal lymphoid cells [16]. In previous studies, mice were orally immunized with the *Lactobacilli plantarum* NC8-pSIP409-HA strain, and this strain induced the production of sIgA, IgG, and HI antibodies, which further induced CD8^+^ T-cell immune response. Most importantly, the oral administration approach provided complete protection against the challenge with mouse-adapted H9N2 virus. These results indicate that *L. plantarum* NC8-pSIP409-HA was more effective in inducing mucosal, humoral, and cellular immune responses [17]. *Lactobacillus acidophilus* has been used to enhance the immunogenicity of Newcastle disease virus (NDV) vaccines. In another study, oral administration of *L. acidophilus* significantly increased the IgG and HI NDV antibody levels in chicks [18]. Probiotics can stimulate the release of cytokines by macrophages and T cells, thereby modulating the mucosal immune response pathways [19,20]. Based on mouse studies, spore adjuvants can induce lung-specific immune responses when delivered as intranasal vaccines [21]. *Bacillus subtilis* spores not only enhance innate immunity for protection against respiratory infections but also induce an increase in antigen-specific antibody and T-cell responses when co-administered with a soluble antigen [22,23]. Probiotics can also be injected intramuscularly together with antigens. This approach involving vaccination with inactivated H9N2 virus together with a *B. subtilis* spore adjuvant in chickens produced a remarkable effect on antigen-specific antibody and T-cell responses against avian influenza virus. It enhanced not only H9N2 virus-specific IgG levels but also CD4^+^ and CD8^+^ T-cell responses, with an increase in proinflammatory cytokine production [24].

In a previous study, the intravenous administration of *Enterococcus faecium* after hyperbaric inactivation induced a substantial increase in nonspecific immune function in mice and restored the normal immune function of immunosuppressed mice [25]. However, presently, there are no reports of intravenous co-administration of bacteria and viruses in a specific ratio. In our preliminary experiment, we found that the intravenous administration of inactivated *E. faecium* alone could not enable mice to resist PRV infection. Therefore, we combined inactivated *E. faecium* with inactivated PRV for intravenous administration to test the experimental results. We expected that this approach could (1) serve as a reference for treating some diseases that cannot be prevented and cured by conventional treatment approaches and (2) function as a model for vaccine development and clinical application.

In the present study, we attempted intravenous co-administration of inactivated *E. faecium* and inactivated PRV to overcome PRV infection in mice. The changes in the levels of immune cells and cytokines in mice were studied to reveal the mechanism underlying the induction of immunity against PRV infection.

## 2. Materials and Methods

### 2.1. Bacterial and Viral Strains and Mice

*E. faecium* CICC 6049 strain was purchased from China Center of Industrial Culture Collection. The PRV Bartha-K61 vaccine strain was obtained from Boehringer Ingelheim Animal Health USA Inc. Specific pathogen free (SPF)-grade Kunming breed mice weighing 18–22 g were purchased from Shandong Taibang Biological Products Co., Ltd. (Tai’an, China).

### 2.2. Preparation of Inactivated E. faecium and Inactivated PRV

The laboratory-preserved *E. faecium* strain was inoculated in a *Lactobacillus* culture medium and incubated at 37 °C for 24 h. The suspension in the culture flask was then centrifuged at 2500 rpm for 6 min; the supernatant was discarded, and the bottom precipitate was retained. The residue was washed three times with 0.9% saline and resuspended with a suitable volume of 0.9% saline to prepare a high concentration of *E. faecium* solution. This solution was placed in an autoclave and sterilized at 121 °C for 15 min and then stored at 4 °C. A suitable volume of the bacterial solution was diluted, and the number of bacterial cells was counted under a microscope by using a THOMA bacterial counting plate. The number of bacterial cells was adjusted to 1.0 × 10^9^ CFU/mL, and the optical density (OD) value was measured at 690 nm. An OD value of 0.38 was assumed as 1× dose, which is the dose required for the experiment.

The solution of PRV Bartha-K61 strain inoculated and amplified on PK-15 cells was harvested, and after centrifugation to remove the sediment, sucrose density gradient centrifugation was performed to obtain ultra-pure PRV Bartha-K61 strain particles. The infectious titers of the viral solution were 10^−7.33^ TCID_50_/0.1 mL. The purified viral solution was inactivated by adding a 40% formaldehyde solution and mixing it thoroughly so that the concentration of the formaldehyde solution used was 0.3% of the viral liquid volume. The inactivated PRV was inoculated into the monolayer of PK-15 cells in three cell culture bottles, cultured at 37 °C, and observed for 7 d before blind transmission of the second generation. No cytopathic effect (CPE) was observed. The inactivated viral solution was inoculated into a bouillon culture medium and a standard nutrient agar and cultured at 37 °C for 24–48 h; no bacterial growth was observed. For safety testing, 10 mice were subcutaneously inoculated with 0.2 mL of the inactivated viral solution and observed for 14 d. A control group without vaccination was also established, and no local or systemic adverse reactions caused by vaccination were observed.

### 2.3. Experimental Design

Animal ethics statements. The present study was conducted in accordance with recommendations from the Guide for the Care and Use of Laboratory Animals of the Ministry of Science and Technology of China. During the experiment, mice showing extreme lethargy were considered moribund and were humanely euthanized.

Sixty 4-week-old mice were divided into 6 groups (10 mice/group) as follows: negative control group (group 1), PRV model group (group 2), inactivated *E. faecium* + inactivated PRV intramuscular injection group (group 3), inactivated *E. faecium* intravenous injection group (group 4), saline + inactivated PRV intravenous injection group (group 5), and inactivated *E. faecium* + inactivated PRV intravenous injection group (group 6). After acclimatization and rearing for 3 days, the mice were treated according to the details provided in Table 1. Three days after antigen inoculation, all the experimental groups, except the negative control group, were administered 20 µL of live PRV containing 10^6.2^ TCID_50_ through a nasal drip, and the negative control group was administered an equal amount of saline nasal drops. Clinical signs, including depression, rough appearance of hair coat, swollen eyes, and neurological symptoms, were monitored daily. All the mice were humanely euthanized at 5 days post challenge (except for the 21 days survival curve experiments).

### 2.4. Survival Curve

After subjecting the mice to immune challenge, they were fed and provided free access to water, and the number of mouse deaths within 21 days was recorded.

### 2.5. Histopathological Examination

During necropsy, the lungs of each group were collected and fixed in 4% phosphate-buffered formalin, embedded in paraffin, cut into 2- to 4-µm-thick slices, and stained with hematoxylin and eosin (H&E). Lesions were observed by an experienced professional pathologist who was blinded to the study groups.

### 2.6. Antibody Analysis

Serum antibodies against the PRV gB protein at 5 days post-infection (DPI) were detected using the porcine pseudorabies virus ELISA antibody detection kit (Wuhan Keqian Biology Co., Ltd., Wuhan, China) in accordance with the manufacturer’s instructions.

### 2.7. Flow Cytometry Analysis

After subjecting them to immune challenge, the mice were fed and provided free access to water for 5 d. After 5 DPI, their spleens were removed and placed in a 24-well plate; 1 mL of RPMI 1640 culture medium was added, mixed, and the culture medium was then discarded. Next, 1 mL of trypsin was injected, and the spleens were cut with sterilized scissors and incubated at 37 °C for approximately 30 min. Subsequently, 1 mL of PBS was added to terminate the digestion. The spleen was grinded, filtered through a cell sieve, and made up to the volume of 10 mL. This suspension was centrifuged at 2500 rpm for 6 min at 4 °C. The supernatant was discarded, and 10 mL of PBS was added to terminate the reaction. Next, the suspension was centrifuged at 4 °C at 2500 rpm for 6 min, the supernatant was discarded, and 1 mL PBS was added. The precipitate was mixed well, filtered through a cell sieve, and divided into two portions. Anti-mouse CD3e FITC-conjugated antibodies, anti-mouse CD4 Alexa Fluor 4700-conjugated antibodies, PE anti-mouse CD8a antibodies, anti-mouse NK1.1 antibodies, and anti-human/mouse CD45R (B220) PerCP-Cyanine 5.5 antibodies were added to approximately 30 µL of the cell suspension. After 15 min of incubation, the samples were subjected to flow cytometry for measurement.

### 2.8. Cytokine Analysis

Sera collected at 5 DPI were used to detect IL-10 levels by using commercial ELISA kits (Mlbio, Inc., Shanghai, China) in accordance with the manufacturer’s instructions. The concentration of cytokines (ng/L) was calculated with a standard curve generated using recombinant mouse cytokines supplied in the kits.

### 2.9. Data Analysis

Kaluza flow cytometric analysis software was used to analyze the results of flow cytometry. GraphPad Prism 9 software was used to prepare graphs. SPSS 23.0 software was used for statistical analysis. One-way analysis of variance (ANOVA) was performed to analyze the significance of the data. Numerical data are expressed as mean ± standard deviation (SD). Different lowercase letters indicate significant differences (*p* < 0.05), while different uppercase letters indicate highly significant differences (*p* < 0.01).

## 3. Results

### 3.1. Clinical Signs and Survival Curve

**Figure 1:**
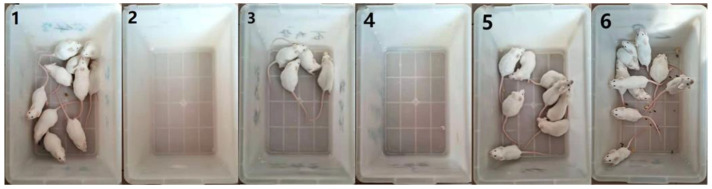
Survival status of mice in each group within 21 days of virus challenge. Group 1 is the negative control group; 2 is the pseudorabies virus model group; 3 is the inactivated *E. faecium* + inactivated pseudorabies virus intramuscular injection group; 4 is the inactivated *E. faecium* intravenous injection group; 5 is the saline + inactivated pseudorabies virus intravenous injection group; 6 is the inactivated *E. faecium* + inactivated pseudorabies virus intravenous injection group.

**Figure 2:**
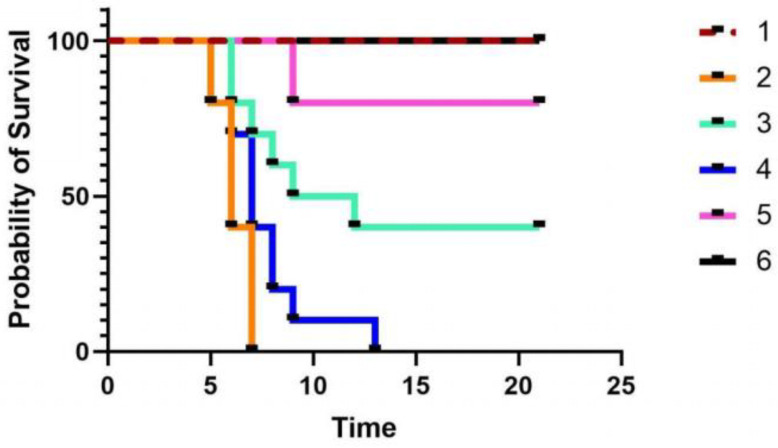
Survival curve of mice from the different groups. Group 1 is the negative control group; 2 is the pseudorabies virus model group; 3 is the inactivated *E. faecium* + inactivated pseudorabies virus intramuscular injection group; 4 is the inactivated *E. faecium* intravenous injection group; 5 is the saline + inactivated pseudorabies virus intravenous injection group; 6 is the inactivated *E. faecium* + inactivated pseudorabies virus intravenous injection group.

Table 2 and Figure 1 and Figure 2 show the results of the number of deaths in the different groups of mice within 21 days after immunization. The mice in each group showed a normal response after immunization, and their appetite for drinking water was normal. Three days after immunization, except for group 1, the remaining groups were challenged with PRV. The mice were normal in the first two days after the virus challenge. From the third day onward, the mice in group 2 and group 4 showed shortness of breath, confusion, loss of appetite, and other clinical symptoms. In the late stage of the disease, the mice were depressed and lost their appetite. Some mice exhibited strong itching symptoms, incomplete body surface hair, and blurred flesh and blood. The mice in group 2 began to die on the 6th day after the virus challenge, and all mice died within 6–8 days. The mice in group 4 began to die on the 6th day after the virus challenge, and all mice died within 6–14 days; this finding confirmed that the inactivated *E. faecium* alone could not resist PRV infection in mice. The mice in group 3 died on the 7th day, and 6 mice died between the 7th and 13th day after the virus challenge. Two mice in group 5 died on the 9th day. Mice in group 6 did not die and showed good growth status, which indicated the advantages of intravenous vaccination. Group 6 showed an excellent immune adjuvant effect of inactivated *E. faecium* as compared to group 5.

### 3.2. Histopathological Examination

**Figure 3:**
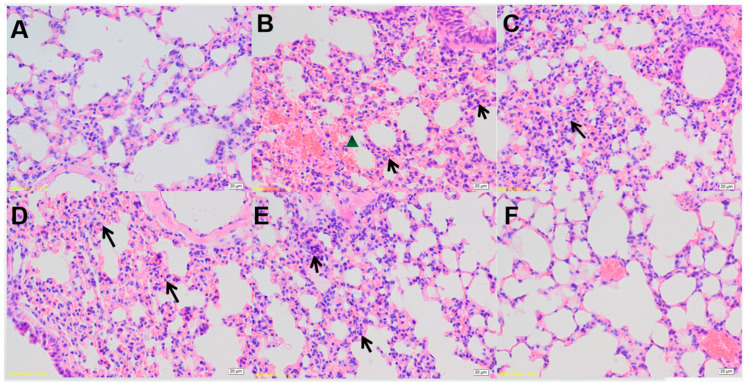
Histopathological changes in the lungs of mice from the different groups. Lung samples from the negative control group (**A**), the pseudorabies virus model group (**B**), the inactivated *E. faecium* + inactivated pseudorabies virus intramuscular injection group (**C**), the inactivated *E. faecium* intravenous injection group (**D**), the saline + inactivated pseudorabies virus intravenous injection group (**E**), and the inactivated *E. faecium* + inactivated pseudorabies virus intravenous injection group (**F**). Tissues were stained with hematoxylin and eosin (magnification 200×). The triangle in the pathological section image represents pulmonary hemorrhage; the arrow shows a widened interstitium with infiltration of interstitial inflammatory cells.

Histopathological lesions in the lungs of different mice groups are shown in Figure 3. Group 1 and group 6 showed no apparent lesions, while the other four treated groups showed varying degrees of pathological changes. Mice in group 2 exhibited pulmonary hemorrhage, interstitial widening, and interstitial inflammatory cell infiltration (Figure 3B). Mice in the remaining three groups showed a widened interstitium with infiltration of interstitial inflammatory cells (Figure 3C–E).

### 3.3. Antibody Analysis

**Figure 4:**
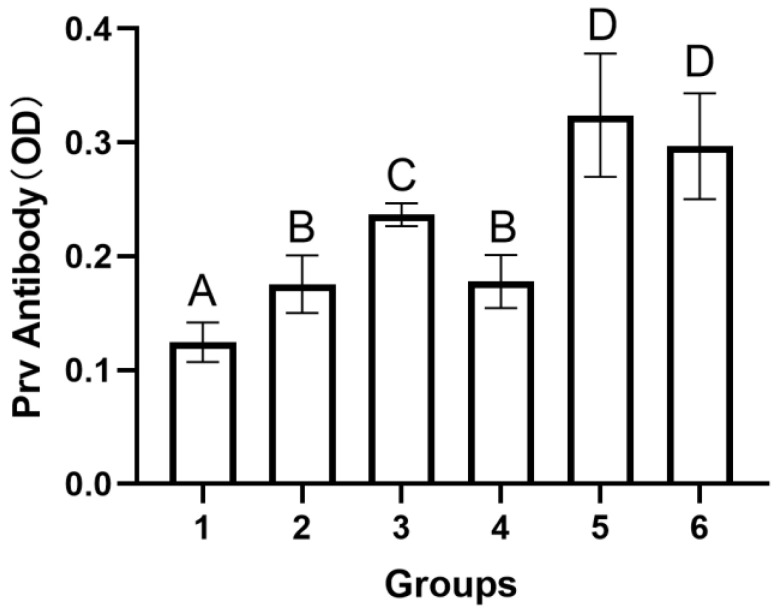
Levels of PRV antibodies in the sera of each group of mice. Serum samples were assayed using the porcine pseudorabies virus ELISA antibody test kit. The secondary antibody in the ELISA kit was replaced with HRP–conjugated sheep anti-mouse IgG to detect PRV antibodies in mouse serum. The dilution of the secondary antibody was 1:5000. The original antibody solution color was replaced with the color of TMB, and the absorbance value was measured at 450 nm. Sera samples were obtained from the negative control group (1), the PRV model group (2), the inactivated *E. faecium* + inactivated PRV intramuscular injection group (3), the inactivated *E. faecium* intravenous injection group (4), the saline + inactivated PRV intravenous injection group (5), and the inactivated *E. faecium* + inactivated PRV intravenous injection group (6). Each point represents mean (±SD) generated from all mice on 5 DPI. Different uppercase letters indicate highly significant differences (*p* < 0.01).

Mice anti-PRV gB antibodies were detected using the porcine PRV ELISA antibody test kit. The secondary antibody in this kit was replaced with the horseradish peroxidase (HRP)–conjugated sheep anti-mouse IgG to determine the amount of PRV antibodies in mouse serum. The dilution of the secondary antibody was 1:5000. As shown in Table 3 and Figure 4, the original antibody solution color was replaced with the color of tetramethylbenzidine (TMB), and the absorbance value was measured at 450 nm. Group 5 and group 6 developed anti-PRV antibodies at 5 DPI. A significant difference was observed between the levels of anti-PRV antibodies of group 5 and group 6 and the remaining groups (*p* < 0.01). No significant difference in the levels of antibodies was observed between group 5 and group 6 (*p* > 0.05). These results show that the intravenous route of vaccination can maximize the production of neutralizing antibodies and play a better role in animal immunity. This finding indicates the feasibility of intravenous vaccine administration for treating PRV infection.

### 3.4. Flow Cytometry Analysis

**Figure 5:**
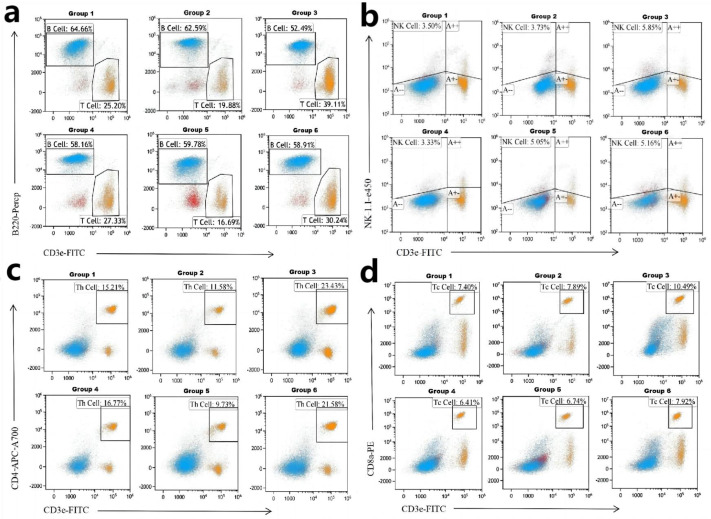
The proportion of each immune cell type in the spleen of mice from the different groups. (**a**–**d**) The proportion of B cells, NK cells, Th cells, and Tc cells, respectively.

As shown in Table 4 and Figure 5, the highest expression and percentage of Th and Tc cells were noted in the spleen of mice from group 3 (5468.9 ± 2443.5 vs. 3577.4 ± 1631.6; 23.43% vs. 10.49%), followed by group 6 (5386.8 ± 1443.9 vs. 2704.5 ± 1182.3; 21.58% vs. 7.92%) (Table 1 and Figure 5c,d). The expression of natural killer (NK) cells in mice spleen was the highest (1462.9 ± 869.4) in group 3 among all the groups (Table 1). These data indicate that the dosage of inactivated *E. faecium* as a vaccine adjuvant is ×10^9^ CFU/mL, 0.1 mL per serving, mixed with antigen 1:1. When the injection route is intramuscular, it has a good activation effect on B cells, Th cells, Tc cells, and NK cells; when the injection route is intravenous, it has a good activation effect on Th cells.

### 3.5. Cytokine Analysis

**Figure 6:**
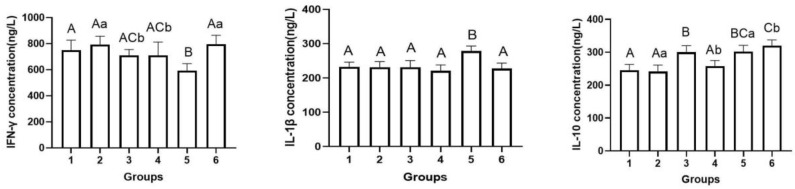
Changes in the expression of cytokines in the serum of mice from the different groups. Group 1 is the negative control group; 2 is the PRV model group; 3 is the inactivated *E. faecium* + inactivated PRV intramuscular injection group; 4 is the inactivated *E. faecium* intravenous injection group; 5 is the saline + inactivated PRV intravenous injection group; 6 is the inactivated *E. faecium* + inactivated PRV intravenous injection group. Different lowercase letters indicate significant differences (*p* < 0.05), while different uppercase letters indicate highly significant differences (*p* < 0.01).

As shown in Figure 6, IFN-γ expression was lower in group 5 than in the other groups (592.22, *p* < 0.0001); however, cytokine IL-1β expression was higher in this group than in the other groups (278.85, *p* < 0.0001). IL-1β expression in group 6 was almost similar to that in group 1 (*p* = 0.498). Group 6 showed the highest expression of cytokine IL-10 (320.08) among all the experimental groups, and a significant difference (*p* = 0.02) in IL-10 expression was noted between this group and group 5. These results show that inactivated *E. faecium* could play an excellent immunomodulatory role of a probiotic when administered as a vaccine adjuvant through the intravenous route. A significant difference (*p* = 0.018) was observed in cytokine IFN-γ expression between group 3 and group 6. Furthermore, cytokine IL-10 expression showed a highly significant difference (*p* = 0.003) between group 3 and group 6. These results indicate that the intravenous route of vaccination was more effective than the intramuscular route in promoting the expression of cytokines in serum when inactivated *E. faecium* was used as a vaccine adjuvant.

## 4. Discussion

The present study showed that inactivated PRV when administered intravenously could reduce the mortality of PRV-infected mice as compared to that when administered intramuscularly. In particular, compared with the saline + inactivated pseudorabies virus intravenous injection, the intravenous administration of inactivated *E. faecium* + inactivated PRV could provide higher protection to mice against PRV infection. This fully reflects the good vaccine adjuvant effect of inactivated *E. faecium*. Thus, this indicates that this study has certain potential research value.

According to a previous report, the BCG vaccine could maximize the protective effect of the vaccine on animals after intravenous administration to rhesus monkeys and mice [26]. In 2020, Darrah et al. obtained the same results experimentally and elucidated the mechanism of action as follows: the cellular immunity of animals plays an important role in immunization; intravenous immunization significantly increases the antibody level in animals; and “trained immunity”-induced epigenetically modified macrophages show enhanced resistance to *Mycobacterium tuberculosis* infection [27]. In the present study, we observed similar results, wherein the mortality rate of mice in the inactivated PRV intravenous injection group after the virus challenge was significantly lower than that in the inactivated PRV intramuscular injection group (Figure 1); furthermore, the level of antibodies produced by mice was significantly higher in the intravenous injection group than in the intramuscular injection group (Figure 2). An ideal vaccination route can reduce animal mortality or substantially reduce clinical signs. The survival curves of mice from the different groups at 21 days after the virus challenge (Figure 2) and the lung pathology of mice from each group (Figure 3) showed that the intravenous administration of inactivated *E. faecium* + inactivated PRV significantly reduced the mortality rate and alleviated the pathological damage caused by virus infection, thus exhibiting an excellent immunization effect. Previous studies have indicated that humoral immunity plays an important secondary role in the regeneration of a specific cellular immune response by the host and is an indispensable component of the organism’s ability to trigger an effective immune response against viral infection [28,29]. In the present experiment, the level of antibodies in the serum of mice in the saline + inactivated PRV intravenous injection group and the inactivated *E. faecium* + inactivated PRV intravenous injection group was significantly higher than that in the remaining experimental groups. This finding indicates the vital effect of intravenous administration of a vaccine on the humoral immunity of animals and also lays the foundation for the rapid generation of specific cellular immunity in animals after the reinvasion of the virus.

Immunomodulation is one of the essential properties of probiotics, and the good immunomodulatory effect of inactivated *E. faecium* can significantly enhance the nonspecific primary and specific immune responses of animals [25]. Similar results were reported by Brisbin et al., thus suggesting that probiotics such as lactic acid bacteria can modulate the innate and adaptive immune responses of animals [30,31,32,33,34,35]. In previous studies, probiotics have been used as adjuvants for oral and intramuscular vaccines for experimental purposes, with some success [7,36,37,38]. The immunomodulatory effect of inactivated *E. faecium* as a vaccine adjuvant was also demonstrated in the present experiment: inactivated *E. faecium*, when co-injected with inactivated PRV as a vaccine adjuvant, significantly increased the number of immune cells such as Th cells, Tc cells, and NK cells in the spleen (Table 4). This in turn had a significant effect on the induction of cellular and humoral immunity and the initial and recovery periods of resistance to the viral infection after the mice were immunized with the vaccine. Thus, inactivated *E. faecium* as a vaccine adjuvant plays a vital role in the production of cellular and humoral immunity and the initial and recovery phases of resistance to viral infection.

In the present study, with regard to serum cytokine levels, mice in the saline + inactivated PRV intravenous injection group showed significantly higher levels of IL-1β than the remaining groups; IL-1β promotes the antigen-presenting ability of antigen-presenting cells (APCs) such as monocytes and macrophages and enhances B cell growth and differentiation and antibody formation. We speculate that the higher antibody levels (OD) in the mice of the saline + inactivated PRV intravenous injection group than in the remaining experimental groups is related to IL-1β concentration. The inactivated *E. faecium* + inactivated PRV intravenous injection group showed significantly higher levels of IFN-γ than the saline + inactivated PRV intravenous injection group. IFN-γ exerts an excellent immunomodulatory effect, regulates the immune functions of T and B lymphocytes, promotes the differentiation of quiescent CD4+ T cells into Th1 cells, promotes the expression of Th1-type cytokines, and enhances cellular immune functions. *E. faecium* as a vaccine adjuvant demonstrated good immunomodulatory effects, as reported earlier by Mohammadi et al. [7,36,37]. IL-10 can promote the survival, expansion, and lethality (cytotoxicity) of CD8^+^ T immune cells in the animal immune system, better promoting long-term protective immunity. The serum IL-10 level was significantly higher in the inactivated *E. faecium* + inactivated PRV intravenous injection group than in the saline + inactivated PRV intravenous injection group, thus demonstrating that *E. faecium* as a vaccine adjuvant exhibited a good immunomodulatory effect; this result was similar to the findings of Mohammadi [7].

Probiotics can influence the immune system of animals through the production of their metabolites, cell wall components, and DNA; furthermore, dead probiotic cells or probiotic-derived ingredients, such as peptidoglycan fragments or DNA, can induce the immunomodulatory effects on animals [39,40]. Even after inactivation, the cytoskeleton of *E. faecium* remains intact; this feature enables to better utilize its probiotic properties. Although probiotics have a high potential and exert good immunomodulatory effects on animals, treatment with probiotics alone does not protect the animal from virus infection. In our study, we found that the administration of inactivated *E. faecium* alone did not enable mice to resist PRV infection; moreover, the inactivated *E. faecium* intravenous injection group and the PRV model group showed no difference in time to onset of death and number of deaths; this result was consistent with the findings of Bavananthasivam et al. [31]. The high survival rate of mice in the inactivated *E. faecium* + inactivated PRV intravenous injection group might contribute to the combination of high humoral immunity produced by intravenous vaccine administration and the fact that inactivated *E. faecium* could adequately stimulate the cellular immunity of the animal.

In the present study, inactivated PRV was used as the antigen, and inactivated *E. faecium* was used as the adjuvant. Inactivated *E. faecium* was co-administered intravenously with the inactivated pathogen. The differences in the protective properties and antibody production in mice following various immunization protocols were compared, and it was concluded that co-administration of inactivated *E. faecium* and the inactivated PRV antigen can provide better protection to animals against PRV infection; this is the innovation aspect of the present study. This study also demonstrates the good immunomodulatory effect of inactivated *E. faecium* and provides new ideas for developing vaccines for infectious diseases and for designing innovative immunization methods.

This study was initiated with the discovery that *E. faecium* is present in the blood of animals [41]. Intravenous administration of *E. faecium* after hyperbaric inactivation led to a significant increase in nonspecific immune function in mice and restored the normal immune function of immunosuppressed mice [25]. Previous studies have shown that an intravenous injection of bacteria or viruses alone can treat tumors with good therapeutic effects [42,43,44,45]. The intravenous administration of the BCG vaccine yielded excellent experimental results as compared to those obtained following other immunization routes, including increased antibody levels in animals, reduced mortality in animals after challenge, and a significant reduction in pathological lesions as observed in autopsy; this confirmed that the BCG vaccine provides the most effective immunization effect on animals when administered intravenously [26,27]. These studies reported that the intravenous administration of bacterial or viral components could exert good immunomodulatory and therapeutic effects on the disease. However, there are no reports on intravenous co-administration of bacterial and viral components in a specific ratio. In the present study, we found that the intravenous injection of inactivated *E. faecium* alone could not enable mice to resist PRV infection. Therefore, we combined inactivated *E. faecium* with inactivated PRV and co-administered them intravenously in mice to test the experimental results. We expect that this approach could serve as a reference to treat some diseases that cannot be prevented and cured by conventional treatment strategies; moreover, this approach could also provide a model for vaccine development and clinical application.

## Figures and Tables

**Table 1:** Immunization methods of mice in each group.

Group	Method of Immunization
Negative control group (group 1)	Intramuscular injection of saline
PRV model group (group 2)	Intramuscular injection of saline
Inactivated *E. faecium* + inactivated PRV intramuscular injection group (group 3)	Intramuscular injection of 1:1 mixture of inactivated *E. faecium* and inactivated PRV 0.2 mL
Inactivated *E. faecium* intravenous injection group (group 4)	Intravenous injection of 0.2 mL of inactivated *E. faecium* injection
Saline + inactivated PRV intravenous injection group (group 5)	Intravenous injection of a 1:1 mixture of saline and inactivated PRV 0.2 mL
Inactivated *E. faecium* + inactivated PRV intravenous injection group (group 6)	Intravenous injection of a 1:1 mixture of inactivated *E. faecium* and inactivated PRV 0.2 mL

**Table 2:** Number of surviving mice in different groups on 21 days.

Groups	Number of Mice
1	10 ^A^
2	0 ^Ba^
3	4 ^BCb^
4	0 ^Ba^
5	8 ^AC^
6	10 ^A^

Group 1 is the negative control group; 2 is the pseudorabies virus model group; 3 is the inactivated *E. faecium* + inactivated pseudorabies virus intramuscular injection group; 4 is the inactivated *E. faecium* intravenous injection group; 5 is the saline + inactivated pseudorabies virus intravenous injection group; 6 is the inactivated *E. faecium* + inactivated pseudorabies virus intravenous injection group. Different lowercase letters indicate significant differences (*p* < 0.05), while different uppercase letters indicate highly significant differences (*p* < 0.01).

**Table 3:** Levels of PRV antibodies in the sera of each group of mice.

Groups	PRV Antibody Concentration (OD450 Value)
1	0.124 ± 0.0172 ^A^
2	0.176 ± 0.0256 ^B^
3	0.237 ± 0.010 ^C^
4	0.178 ± 0.023 ^B^
5	0.324 ± 0.054 ^D^
6	0.297 ± 0.046 ^D^

Group 1 is the negative control group; 2 is the PRV model group; 3 is the inactivated *E. faecium* + inactivated PRV intramuscular injection group; 4 is the inactivated *E. faecium* intravenous injection group; 5 is the saline + inactivated PRV intravenous injection group; 6 is the inactivated *E. faecium* + inactivated PRV intravenous injection group. Different uppercase letters indicate highly significant differences (*p* < 0.01).

**Table 4:** The number of each immune cell type in the spleen of mice from the different groups.

Groups	B Cell Count (pcs)	Th Cell Count (pcs)	Tc Cell Count (pcs)	NK Cell Count (pcs)
1	17,376.9 ± 7048.6 ^a^	4055.11 ± 1130.6	2005.2 ± 593.1 ^A^	1125.8 ± 367.6
2	14,265.8 ± 4364.1	2800.10 ± 529.7 ^A^	2004.7 ± 622.1 ^A^	885.7 ± 412.6 ^a^
3	17,496.5 ± 7015.1 ^a^	5468.9 ± 2443.5 ^Bb^	3577.4 ± 1631.6 ^B^	1462.9 ± 869.4 ^Bb^
4	11,947 ± 3757.2 ^b^	3140 ± 1105.5 ^A^	1807.8 ± 1051.6 ^A^	812.7 ± 189.1 ^Aa^
5	15,034.7 ± 5906	3867.1 ± 1665.4 ^a^	2099 ± 969.1 ^A^	1283.4 ± 511.7 ^ABb^
6	15,645.5 ± 6016	5386.8 ± 1443.9 ^Bb^	2704.5 ± 1182.3	1070.1 ± 404.5

Group 1 is the negative control group; 2 is the PRV model group; 3 is the inactivated *E. faecium* + inactivated PRV intramuscular injection group; 4 is the inactivated *E. faecium* intravenous injection group; 5 is the saline + inactivated PRV intravenous injection group; 6 is the inactivated *E. faecium* + inactivated PRV intravenous injection group. Different lowercase letters indicate significant differences (*p* < 0.05), while different uppercase letters indicate highly significant differences (*p* < 0.01).

## Data Availability

Not applicable.
